# An orphan kinesin in *Trypanosoma brucei* regulates hook complex assembly and Golgi biogenesis

**DOI:** 10.1128/mbio.02634-24

**Published:** 2024-10-30

**Authors:** Qing Zhou, Yasuhiro Kurasawa, Huiqing Hu, Thiago Souza Onofre, Ziyin Li

**Affiliations:** 1 Department of Microbiology and Molecular Genetics, McGovern Medical School, University of Texas Health Science Center at Houston, Houston, Texas, USA; Washington University in St. Louis School of Medicine, St. Louis, Missouri, USA

**Keywords:** *Trypanosoma brucei*, centrin arm, hook complex, kinesin, Golgi

## Abstract

Kinesins are microtubule-based motor proteins that play diverse cellular functions by regulating microtubule dynamics and intracellular transport in eukaryotes. The early branching kinetoplastid protozoan *Trypanosoma brucei* has an expanded repertoire of kinetoplastid-specific kinesins and orphan kinesins, many of which have unknown functions. We report here the identification of an orphan kinesin named KIN-G that plays an essential role in maintaining hook complex integrity and promoting Golgi biogenesis in *T. brucei*. KIN-G localizes to the distal portion of the centrin arm of the flagellum-associated hook complex through association with the centrin arm protein TbCentrin4. Knockdown of KIN-G in *T. brucei* disrupts the integrity of the hook complex by reducing the length of the centrin arm and eliminating the shank part of the hook complex, thereby impairing flagellum attachment zone elongation and flagellum positioning, which leads to unequal cytokinesis. KIN-G associates with Golgi through a centrin arm-localized Golgi peripheral protein named CAAP1, which maintains Golgi-centrin arm association to facilitate Golgi biogenesis. Knockdown of KIN-G impairs Golgi biogenesis by disrupting CAAP1 at the centrin arm, thereby impairing the maturation of centrin arm-associated Golgi. *In vitro* microtubule gliding assays demonstrate that KIN-G is a plus end-directed motor protein, and its motor activity is required for hook complex assembly and Golgi biogenesis. Together, these results identify a kinesin motor protein for promoting hook complex assembly and uncover a control mechanism for Golgi biogenesis through KIN-G-mediated maintenance of Golgi-hook complex association.

**IMPORTANCE:**

*Trypanosoma brucei* has a motile flagellum, which controls cell motility, cell morphogenesis, cell division, and cell-cell communication, and a set of cytoskeletal structures, including the hook complex and the centrin arm, associates with the flagellum. Despite the essentiality of these flagellum-associated cytoskeletal structures, their mechanistic roles and the function of their associated proteins remain poorly understood. Here, we demonstrate that the orphan kinesin KIN-G functions to promote the biogenesis of the hook complex and the Golgi apparatus. KIN-G exerts this function by mediating the association between centrin arm and Golgi through the centrin arm protein TbCentrin4 and a novel Golgi scaffold protein named CAAP1, thereby bridging the two structures and maintaining their close association to facilitate the assembly of the two structures. These findings uncover the essential involvement of a kinesin motor protein in regulating the biogenesis of the hook complex and the Golgi in trypanosomes.

## INTRODUCTION

*Trypanosoma brucei* is a unicellular parasite causing sleeping sickness in humans and nagana in livestock, posing severe threats to human health and economic development in sub-Saharan Africa. The parasite has a motile flagellum, which is nucleated from the basal body, exits the cell body through the flagellar pocket, and attaches to the cell membrane *via* a specialized cytoskeletal structure termed the flagellum attachment zone (FAZ). In the flagellar pocket region, the flagellum is associated with several specialized cytoskeletal structures, including the hook complex (1) and the flagellar pocket collar (FPC) (2), with the horseshoe-shaped FPC wrapping around the flagellum and the hook complex sitting atop the FPC (1). The hook complex has a hairpin-like morphology and is composed of a fishhook-like structure and a centrin arm structure, which sits alongside the shank part of the fishhook (1, 3). The microtubule quartet (MtQ) refers to a specialized set of four microtubules that originate between the mature basal body and the pro-basal body, wrap around the flagellar pocket, pass through the FPC and the hook complex, and run alongside the intracellular FAZ filament to extend to the distal tip of the cell body (1, 4). The proximal end of the intracellular FAZ filament is embedded between the shank part of the fishhook and the centrin arm (1), and it appears that maintenance of hook complex integrity is critical for FAZ elongation and flagellum positioning (5, 6).

The procyclic (insect) form of trypanosomes has a single Golgi apparatus, which undergoes *de novo* duplication, with the new Golgi being assembled next to the ER exit site (ERES) and next to the old Golgi (7). The Golgi is located in close proximity to the centrin arm (or the bi-lobed structure) of the hook complex, and its duplication is dependent on the centrin arm protein TbCentrin2 (8). In cells depleted of TbCentrin2, new Golgi is not formed (8); therefore, the centrin arm appears to determine the site where new Golgi is assembled. Golgi biogenesis in trypanosomes also requires the Polo-like kinase homolog TbPLK, which localizes to the centrin arm and other subcellular structures (9). However, unlike TbCentrin2 knockdown, depletion of TbPLK in trypanosomes exerts opposite effects on Golgi biogenesis. Instead of inhibiting the assembly of new Golgi, TbPLK knockdown produces cells containing numerous smaller Golgi, most of which do not associate with the mal-formed centrin arm (9). The underlying mechanisms for the distinct roles of TbPLK and TbCentrin2 in Golgi biogenesis remain unknown, but it may be attributed to the distinctive effects of their knockdown on the centrin arm, with TbCentrin2 knockdown completely eliminating the centrin arm structure (6) and TbPLK knockdown disrupting the integrity of the centrin arm (9).

We previously identified a hook complex-localized orphan kinesin, named KIN-G (10), implying the potential involvement of kinesin-mediated activities for hook complex-related functions. Kinesins are microtubule-based motor proteins, which regulate microtubule dynamics and mediate intracellular transport for numerous cellular regulations in eukaryotes (11). The trypanosome genome encodes an unexpectedly large number of orphan kinesins and kinetoplastid-specific kinesins (12), some of which have been functionally characterized and were found to localize to distinct subcellular structures and play diverse cellular functions (13–21). In this report, we characterized KIN-G in the procyclic form of *T. brucei* and identified its roles in promoting hook complex assembly and regulating Golgi biogenesis. We also showed that KIN-G is required for FAZ elongation and flagellum positioning, thereby promoting cell division plane placement to ensure symmetrical cytokinesis in *T. brucei*. These findings discovered a novel function of a kinesin in regulating hook complex assembly and Golgi biogenesis and further highlighted the diverse cellular functions played by orphan kinesins in trypanosomes.

## RESULTS

### KIN-G localizes to the distal part of the centrin arm

**Fig 1:**
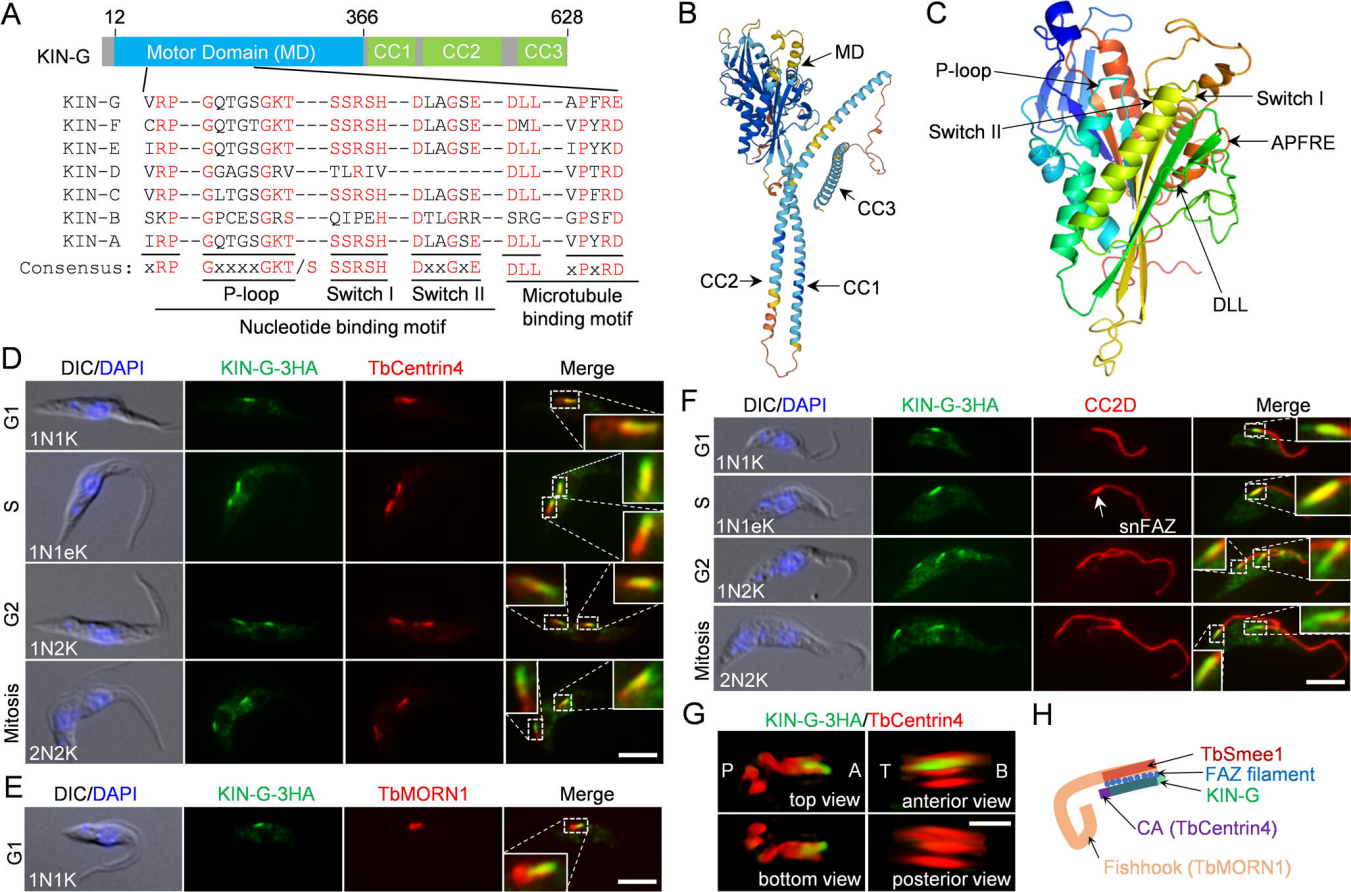
Structural prediction and subcellular localization of KIN-G. (**A**). Schematic drawing of the structural motifs in KIN-G. MD: Motor domain; CC: coiled coil. (**B**). Prediction of KIN-G structure by AlphaFold. The locations of structural domains are indicated. (**C**). Structural modeling of the KIN-G motor domain by SWISS-MODEL. The locations of the motifs involved in nucleotide binding and microtubule binding are indicated. The template used is 6A20, the rat kinesin-3 family protein KIF13B. (**D**). Co-localization of 3HA-tagged KIN-G and TbCentrin4 during the cell cycle. The insets show the 3× zoom-in view of the co-localization of KIN-G and TbCentrin4. Scale bar: 5 µm. (**E**). Co-immunostaining of KIN-G-3HA and TbMORN1. The inset shows the 3× zoom-in view of the co-localization of KIN-G and TbMORN1. Scale bar: 5 µm. (**F**). Co-immunostaining of KIN-G-3HA and CC2D, which labels the intracellular FAZ filament. Insets show the 3× zoom-in view of the co-localization of KIN-G and the proximal end of the FAZ. snFAZ: short, new FAZ. Scale bar: 5 µm. (**G**). 3D-SIM super-resolution microscopic analysis of the co-localization of KIN-G-3HA and TbCentrin4. P: posterior; A: anterior; T: top; B: bottom. Scale bar: 0.5 µm. (**H**). Schematic drawing of the relative localization of KIN-G, hook complex components (TbCentrin4, TbMORN1, and TbSmee1), and the FAZ filament.

We recently determined the subcellular localization of 21 kinetoplastid-specific kinesins and orphan kinesins in *T. brucei* (10), and one orphan kinesin (Tb927.6.1770), which we named KIN-G, localizes to a subcellular structure near the hook complex. In this work, we characterized KIN-G for its precise subcellular localization and cellular functions. KIN-G contains an N-terminal motor domain (MD), which comprises a conserved nucleotide-binding motif and a microtubule-binding motif, and three coiled-coil motifs at its C-terminus (Fig. 1A through C), as predicted by AlphaFold (22, 23) and SWISS-MODEL (24, 25). To determine the precise location of KIN-G within the hook complex, we endogenously tagged KIN-G with a triple HA epitope and performed co-immunofluorescence microscopy with the anti-TbCentrin4 antibody and the anti-TbMORN1 antibody as markers for the centrin arm structure and the fishhook-like structure of the hook complex, respectively (Fig. 1D and E). KIN-G fluorescence signal displayed a bar-shaped morphology, located to the centrin arm and overlapping with the shank part of the fishhook (Fig. 1D and E). In G1-phase (1N1K, N: nucleus; K: kinetoplast) cells, KIN-G was localized to the entire centrin arm (Fig. 1D). From S-phase (1N1eK cells, eK: elongated kinetoplast) to mitotic phase (2N2K cells), KIN-G appeared to concentrate at the distal part of the new centrin arm and additionally extended beyond the distal end of the new centrin arm, but it overlapped almost entirely with the old centrin arm (Fig. 1D).

Since the proximal end of the FAZ filament is embedded between the centrin arm and the shank part of the fishhook (1), we examined whether KIN-G overlaps with the proximal end of the FAZ filament by co-immunostaining KIN-G-3HA and the FAZ filament protein CC2D. KIN-G was found to overlap partly with the proximal end of the FAZ filament in G1 (1N1K) cells, almost entirely with the growing new FAZ filament in S-phase (1N1eK) cells, and with the proximal ends of both the new and the old FAZ filaments in G2 (1N2K) and mitotic (2N2K) cells (Fig. 1F). The localization of KIN-G to the distal part of the centrin arm and, particularly, to the growing new FAZ filament during the S-phase of the cell cycle suggests potential roles of KIN-G in controlling the duplication of the hook complex and in regulating the assembly of the new FAZ filament.

The localization of KIN-G to the distal part of the centrin arm was further confirmed by three-dimensional structured illumination microscopy (3D-SIM) super-resolution microscopy (Fig. 1G). The relative locations of KIN-G, some of the known hook complex components, TbCentrin4, TbMORN1, and TbSmee1, and the intracellular FAZ filament were depicted in Fig. 1H.

### Knockdown of KIN-G causes defective cytokinesis

**Fig 2:**
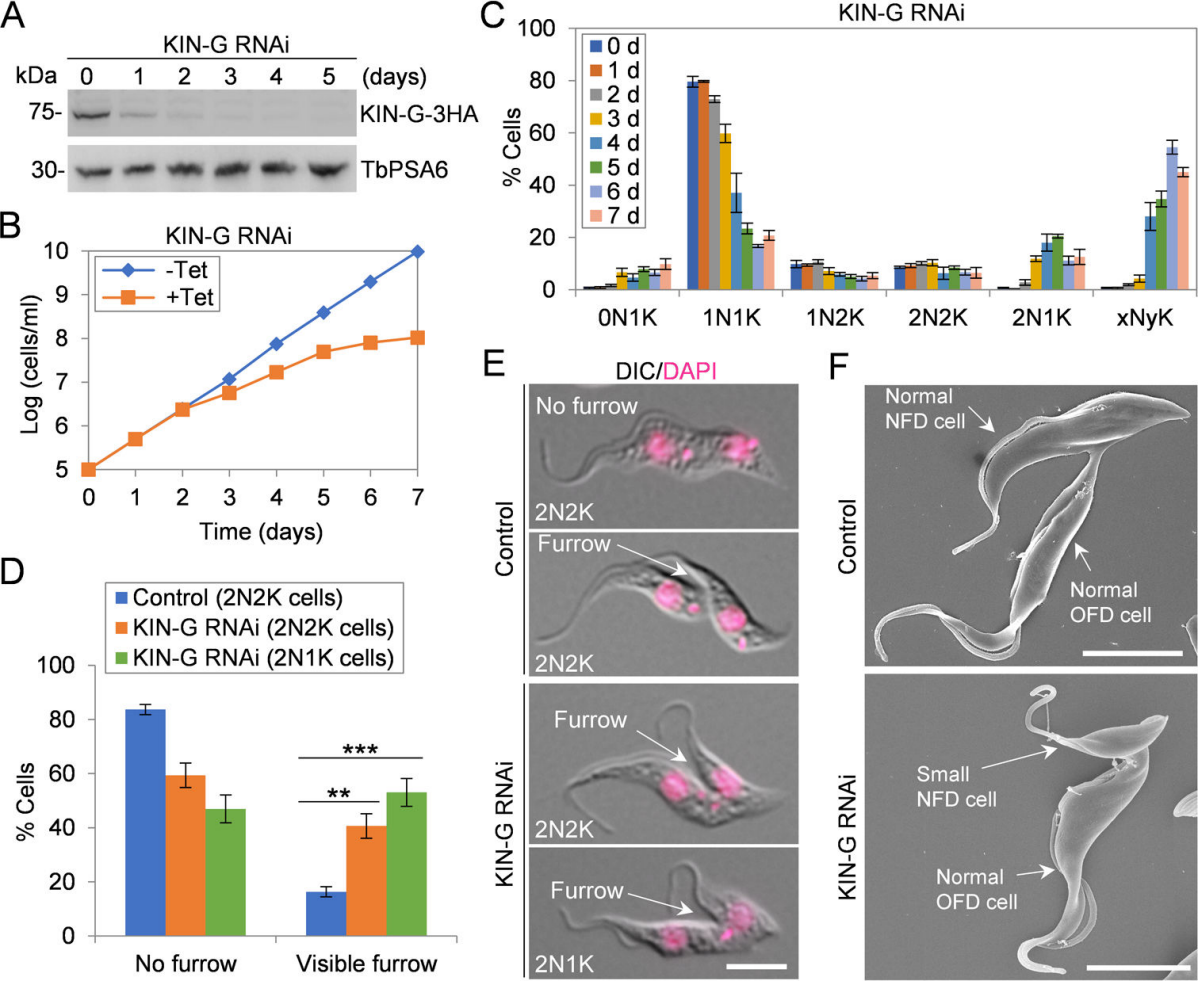
Knockdown of KIN-G in procyclic trypanosomes causes unequal cytokinesis. (**A**). Knockdown of KIN-G by RNAi. Endogenous KIN-G-3HA was detected by the anti-HA antibody. TbPSA6 served as the loading control. (**B**). KIN-G RNAi caused growth defects in procyclic trypanosomes. (**C**). Effect of KIN-G RNAi on cell cycle progression. Shown is the counting of cells with different numbers of nucleus (**N**) and kinetoplast (**K**). 100 cells each were counted, and error bars indicate SD from three independent experiments. (**D**). Effect of KIN-G RNAi on cleavage furrow ingression. Shown is the percentage of the bi-nucleated cells with or without a visible cleavage furrow. 100 cells each were counted and error bars indicate SD from three independent experiments. **P < 0.01; ***P < 0.001. (**E**). Control and KIN-G RNAi cells prior to and during cytokinesis. Scale bars: 5 µm. (**F**). Scanning electron microscopic analysis of dividing control and KIN-G RNAi cells, showing asymmetrical cytokinesis in a KIN-G RNAi cell. NFD: new flagellum daughter; OFD: old flagellum daughter. Scale bar: 5 µm.

We performed RNAi to investigate the function of KIN-G in the procyclic form of *T. brucei*. To examine the efficiency of RNAi, western blotting was carried out to detect the protein level of KIN-G, which was endogenously tagged with a triple HA epitope in the KIN-G RNAi cell line, before and after RNAi induction. KIN-G protein level was gradually decreased after 1 day of RNAi induction, although it was not completely depleted after 3 days of RNAi (Fig. 2A). This downregulation of KIN-G by RNAi caused growth defects after RNAi induction for 3 days, resulting in the increase of the doubling time from ~12 h to ~24 h (Fig. 2B). We next analyzed the effects of KIN-G RNAi on cell cycle progression by counting the cells with different numbers of nuclei (N) and kinetoplasts (K) because cells at different cell cycle stages contain different numbers of nuclei and kinetoplasts. Cells at the G1 phase and early S-phase have one nucleus and one kinetoplast (1N1K), cells at the cell cycle stages from late S-phase to early anaphase have one nucleus and two kinetoplasts (1N2K), and cells at the cell cycle stages from late anaphase to cytokinesis have two nuclei and two kinetoplasts (2N2K). After RNAi induction, the number of 1N1K cells gradually decreased, whereas the number of xNyK (x > 2, y ≥ 1) cells gradually increased and accumulated (>28%) after RNAi induction for 4 days (Fig. 2C), demonstrating impaired cytokinesis. Notably, other abnormal cell types, such as 2N1K cells and 0N1K cells, also accumulated after KIN-G RNAi (Fig. 2C). These 2N1K and 0N1K cells could be derived from aberrant cytokinesis of 2N2K cells. The 2N1K cells could also be generated from 1N1K cells following normal nuclear division but inhibited kinetoplast duplication/segregation. Nonetheless, after KIN-G RNAi for 72 h, bi-nucleated (2N2K and 2N1K) cells were increased from ~8% to 22% (Fig. 2C), further demonstrating impaired cytokinesis. Since the earliest time point for KIN-G RNAi to show growth defects was at day 3, all the subsequent phenotypic analyses were performed at day 3 of RNAi induction.

**Fig 3:**
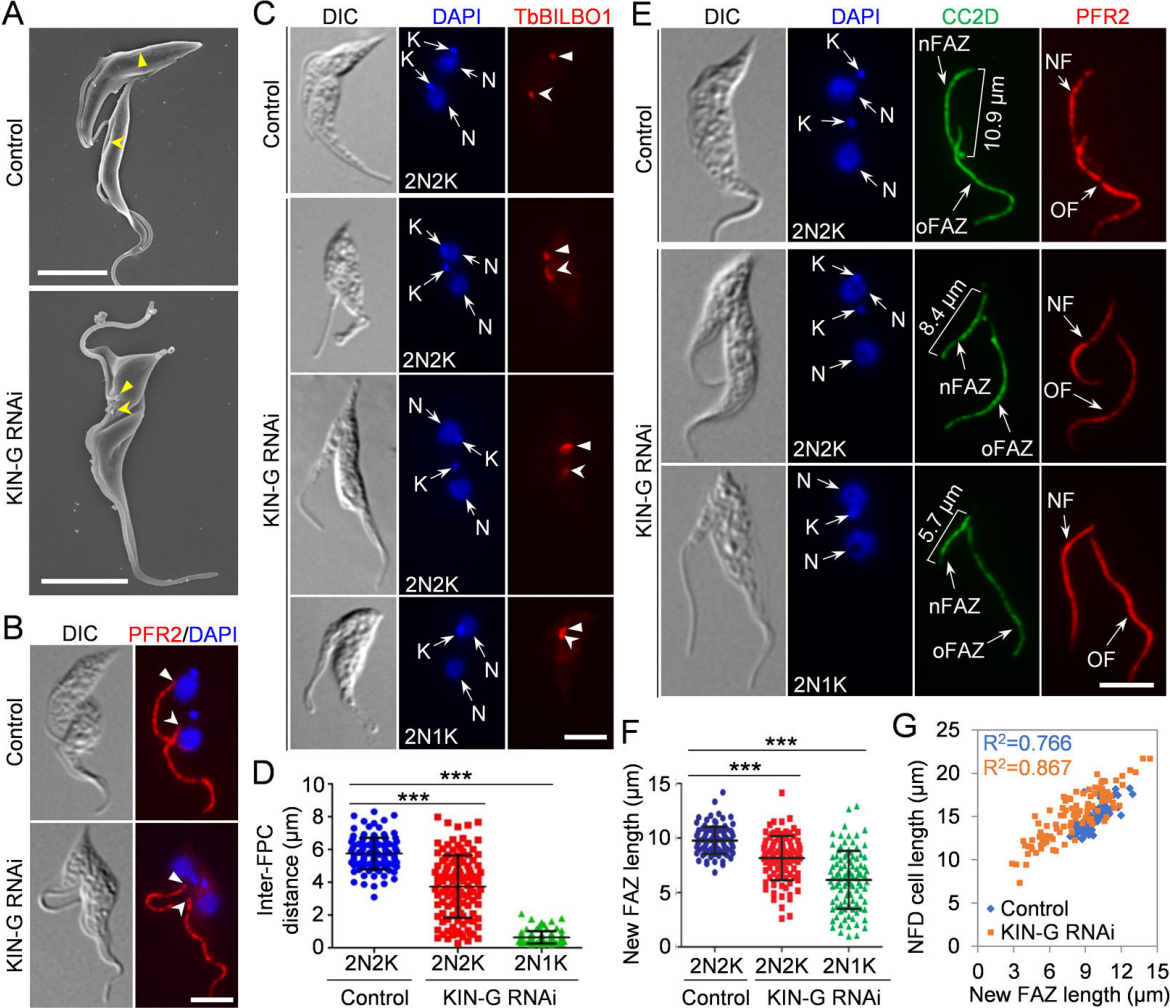
Knockdown of KIN-G disrupts flagellum positioning and FAZ elongation. (**A**). Scanning electron microscopy to examine the flagellum in control and KIN-G RNAi cells. Solid and open arrowheads indicate the positions of the proximal base of the new and the old flagella, respectively. Scale bars: 5 µm. (**B**). Immunofluorescence microscopy with the anti-PFR2 antibody (clone L8C4) to examine the flagellum in control and KIN-G RNAi cells. Solid and open arrowheads indicate the locations of the proximal base of the new and the old flagella. Scale bar: 5 µm. (**C**). Immunostaining of the flagellar pocket collar (FPC) with the anti-TbBILBO1 antibody in control and KIN-G RNAi cells. Solid and open arrowheads indicate the locations of the new and the old FPCs, respectively. Scale bar: 5 µm. (**D**). Measurement of the inter-FPC distance in bi-nucleated cells of control and KIN-G RNAi. 100 cells each were used for measurement. ***P < 0.001. (**E**). Co-immunostaining of the FAZ filament by the anti-CC2D antibody and the flagellum by the anti-PFR2 antibody. nFAZ: new FAZ; oFAZ: old FAZ; NF: new flagellum; OF: old flagellum. Scale bar: 5 µm. (**F**). Measurement of the length of the new FAZ in bi-nucleated cells before and after KIN-G RNAi. 100 cells each were used for measurement. ***P < 0.001. (**G**). Measurement of the length of the new-flagellum daughter (NFD) cell and its correlation with the length of the new FAZ. 100 cells each were counted.

The bi-nucleated (2N2K and 2N1K) cells that contain a visible cleavage furrow were significantly increased after KIN-G RNAi induction for 72 h (Fig. 2D and E). Notably, many of the bi-nucleated cells from the KIN-G RNAi-induced population that are undergoing cytokinesis appeared to produce a smaller-sized new flagellum daughter (NFD) cell containing a longer unattached flagellum and a normal-sized old-flagellum daughter (OFD) cell containing a normally attached flagellum (Fig. 2F). This is in contrast to the control cells, in which the NFD cell of a dividing bi-flagellated cell had a normally attached flagellum and a cell body size similar to that of the OFD cell (Fig. 2F). It should be noted that in wild-type trypanosomes, the NFD cell and the OFD cell of a dividing cell are not identical in size (26); thus, cytokinesis in wild-type trypanosome cells is not absolutely symmetrical. However, the NFD cell of the dividing KIN-G RNAi cell was much smaller than the NFD cell of the dividing control cell (Fig. 2F) and had a shorter FAZ and a longer unattached flagellum (Fig. 2F and see Fig. 3). These results suggest that knockdown of KIN-G causes defective cytokinesis, likely by impairing the placement of the cell division plane.

### KIN-G is required for flagellum positioning and FAZ elongation

The hook complex is one of the flagellum-associated cytoskeletal structures implicated in regulating the segregation of the newly assembled flagellum (5). Since KIN-G localizes to the distal part of the centrin arm (Fig. 1D), we examined the potential effect of KIN-G knockdown on the segregation of the newly assembled flagellum. Scanning electron microscopic analysis showed that KIN-G RNAi cells were able to assemble a new flagellum, but the newly assembled flagellum was positioned in close proximity to the old flagellum (Fig. 3A), suggesting defective segregation and positioning of the new flagellum. Immunofluorescence microscopy with the antibody against the paraflagellar rod protein PFR2 confirmed the defect in flagellum segregation and positioning after KIN-G RNAi (Fig. 3B). In addition, immunofluorescence microscopy with the antibody against the FPC protein TbBILBO1 showed that segregation of the new FPC was also impaired after KIN-G RNAi (Fig. 3C and D), further confirming that depletion of KIN-G disrupts flagellum segregation. Altogether, these results demonstrated the requirement of KIN-G for the segregation/positioning of the newly assembled flagellum.

The production of a small-sized NFD cell with a longer unattached flagellum by KIN-G RNAi (Fig. 2E and F) led us to hypothesize that KIN-G RNAi disrupted the elongation of the new FAZ. To test this hypothesis, we co-immunostained the control and KIN-G RNAi cells with the anti-CC2D antibody, which labels the intracellular FAZ filament (27), and the anti-PFR2 antibody, which labels the flagellum (28), and measured the length of the new FAZ and the unattached new flagellum in control and KIN-G RNAi cells. We focused on bi-nucleated cells, as the flagellum and the FAZ in these cells should have already been fully synthesized; therefore, this allows an accurate examination of the effect of KIN-G RNAi on the assembly of the new FAZ. The results showed that the length of the new FAZ of the bi-nucleated cells was reduced from an average of ~9.8 µm (2N2K cells, control) to ~8.2 µm (2N2K cells, RNAi) and ~6.2 µm (2N1K cells, RNAi) (Fig. 3E and F). Therefore, the knockdown of KIN-G inhibited the elongation of the new FAZ.

Finally, we measured the length of the NFD cell of dividing bi-nucleated cells and analyzed the correlation between cell length and FAZ length. We found that the small-sized NFD cells all possessed a short FAZ, and there was a positive correlation between the NFD cell length and the new FAZ length (Fig. 3G), suggesting that the length of the new FAZ determines the size of the NFD cell, likely by determining the position of the cell division plane, as having been demonstrated previously (27). Therefore, the unequal cytokinesis observed in KIN-G RNAi cells (Fig. 2E and F) is likely attributed to the mis-positioning of the cell division plane caused by the defective elongation of the new FAZ (Fig. 3E and F).

### Knockdown of KIN-G disrupts the integrity of the hook complex

**Fig 4:**
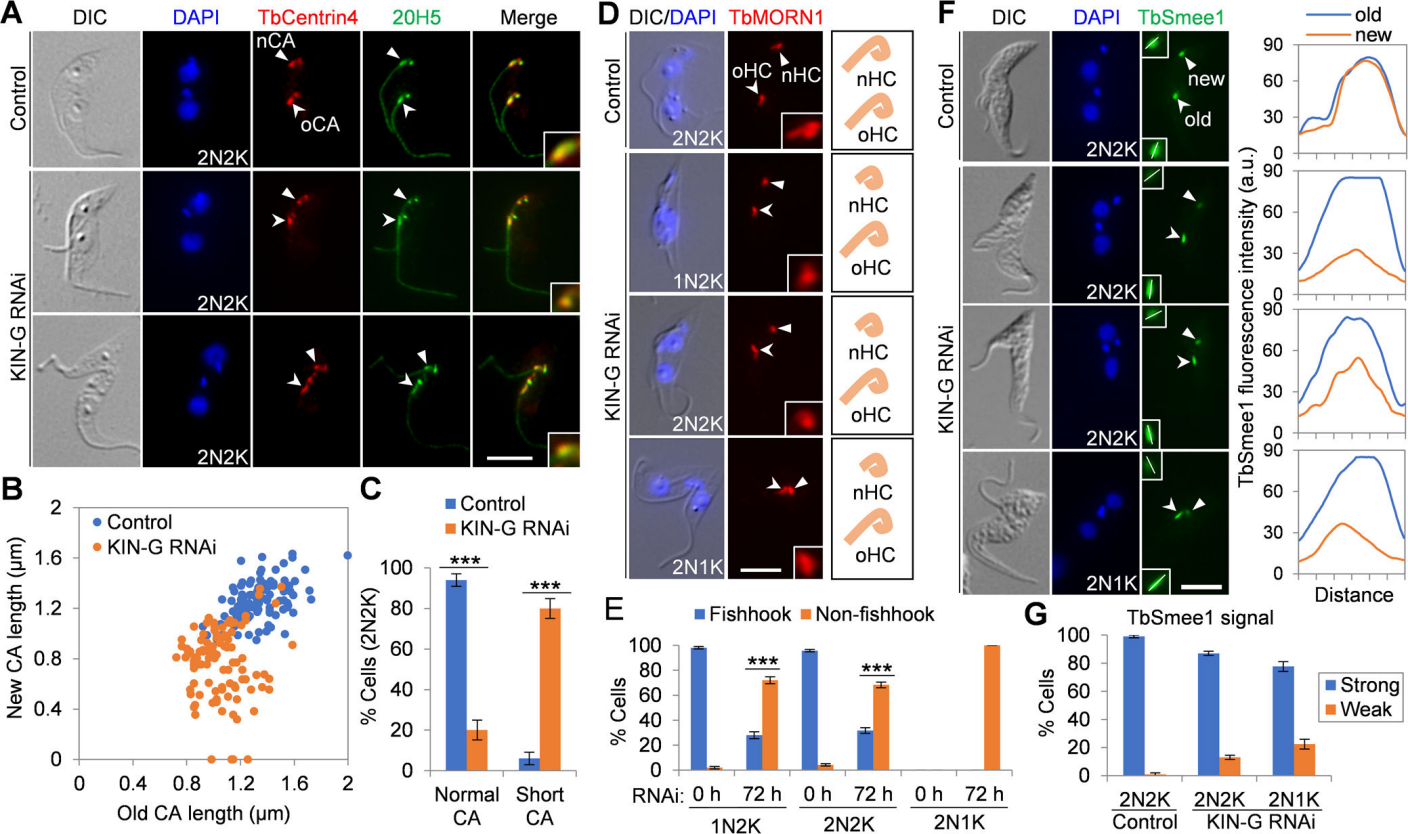
KIN-G is required for hook complex assembly. (**A**). Immunofluorescence microscopy to examine the centrin arm with the anti-TbCentrin4 antibody and the pan-centrin antibody 20H5. Solid arrowheads indicate the new centrin arm, whereas open arrowheads indicate the old centrin arm. The insets show the 3× zoom-in view of the new centrin arm. Scale bar: 5 µm. (**B**). Measurement of the length of the new and old centrin arm structures of control and KIN-G RNAi cells. 100 cells each were used for measurement. (**C**). Quantitation of 2N2K cells with the new centrin arm of normal length or short length before and after KIN-G RNAi for 72 h. The cut-off for normal and short new centrin arm is 1.0 µm. 100 cells each were counted and error bars indicate SD from three independent experiments. ***P < 0.001. (**D**). Immunostaining of the hook complex by the anti-TbMORN1 antibody. Solid arrowheads indicate the new hook complex (nHC), whereas open arrowheads indicate the old hook complex (oHC). The insets show the 3× zoom-in view of the nHC. The cartoon on the right depicts the morphology of nHC and oHC. Scale bars: 5 µm. (**E**). Percentage of cells with the new hook complex displaying a typical fishhook-like shape or a non-fishhook-like shape in control and KIN-G RNAi cells. 100 cells each were counted, and error bars indicate SD from three independent experiments. ***P < 0.001. (**F**). Effect of KIN-G knockdown on the centrin arm-associated protein TbSmee1, which was endogenously tagged with a triple HA epitope. The insets show the 3× zoom-in view of the TbSmee1 signal at the new and the old hook complexes, and the white line indicates the transect for quantitative measurement of the TbSmee1 signal shown at the right panel. Scale bar: 5 µm. (**G**). Quantitation of bi-nucleated cells with strong or weak TbSmee1-3HA fluorescence signal before and after KIN-G RNAi. 100 cells each were counted, and error bars indicate SD from three independent experiments. ***P < 0.001.

The localization of KIN-G to the distal part of the centrin arm (Fig. 1D) prompted us to investigate the potential role of KIN-G in regulating centrin arm assembly. To this end, we immunostained cells with the anti-TbCentrin4 antibody and the pan-centrin antibody 20H5, and then examined the centrin arm in bi-nucleated cells before and after KIN-G RNAi. Knockdown of KIN-G significantly reduced the length of the new centrin arm (Fig. 4A and B). Cells with the length of the new centrin arm longer than 1.0 µm were reduced from ~94% to ~20%, whereas cells with the length of the new centrin arm shorter than 1.0 µm were increased from ~6% to ~80% after KIN-G RNAi for 72 h (Fig. 4C). These results suggest that KIN-G is required to maintain the integrity of the centrin arm. Because the centrin arm is associated with the shank part of the fishhook (1), we wondered whether the knockdown of KIN-G also disrupts the integrity of the fishhook. To this end, we immunostained cells with the anti-TbMORN1 antibody to label the fishhook. The old fishhook in KIN-G RNAi cells maintained the fishhook-like morphology, but the new fishhook in KIN-G RNAi cells appeared to lose the shank part in ~72% of the 1N2K cells, ~68% of the 2N2K cells, and all (100%) of the 2N1K cells (Fig. 4D and E), suggesting the impairment of the fishhook structure of the hook complex. Altogether, these results demonstrated the requirement of KIN-G for promoting the assembly of the hook complex.

We next asked whether the impairment of hook complex integrity affects the recruitment of TbSmee1, which localizes to the shank part of the fishhook next to the centrin arm (1), and CAAP1 (Centrin Arm-Associated Protein 1), which was previously reported to localize to the centrin arm (6, 29). We endogenously tagged TbSmee1 and CAAP1 with a C-terminal triple HA epitope in the KIN-G RNAi cell line and then analyzed their localization by immunofluorescence microscopy. Knockdown of KIN-G caused a reduction in the intensity of the TbSmee1 signal at the new hook complex of bi-nucleated cells, as demonstrated by the measurement of the TbSmee1 signal intensity with ImageJ (Fig. 4F). However, the reduction in TbSmee1 signal intensity in the new hook complex occurred in ~13% of the 2N2K cells and ~22% of the 2N1K cells (Fig. 4G), suggesting that the defects in hook complex integrity only moderately affect TbSmee1.

### KIN-G is required for Golgi biogenesis

**Fig 5:**
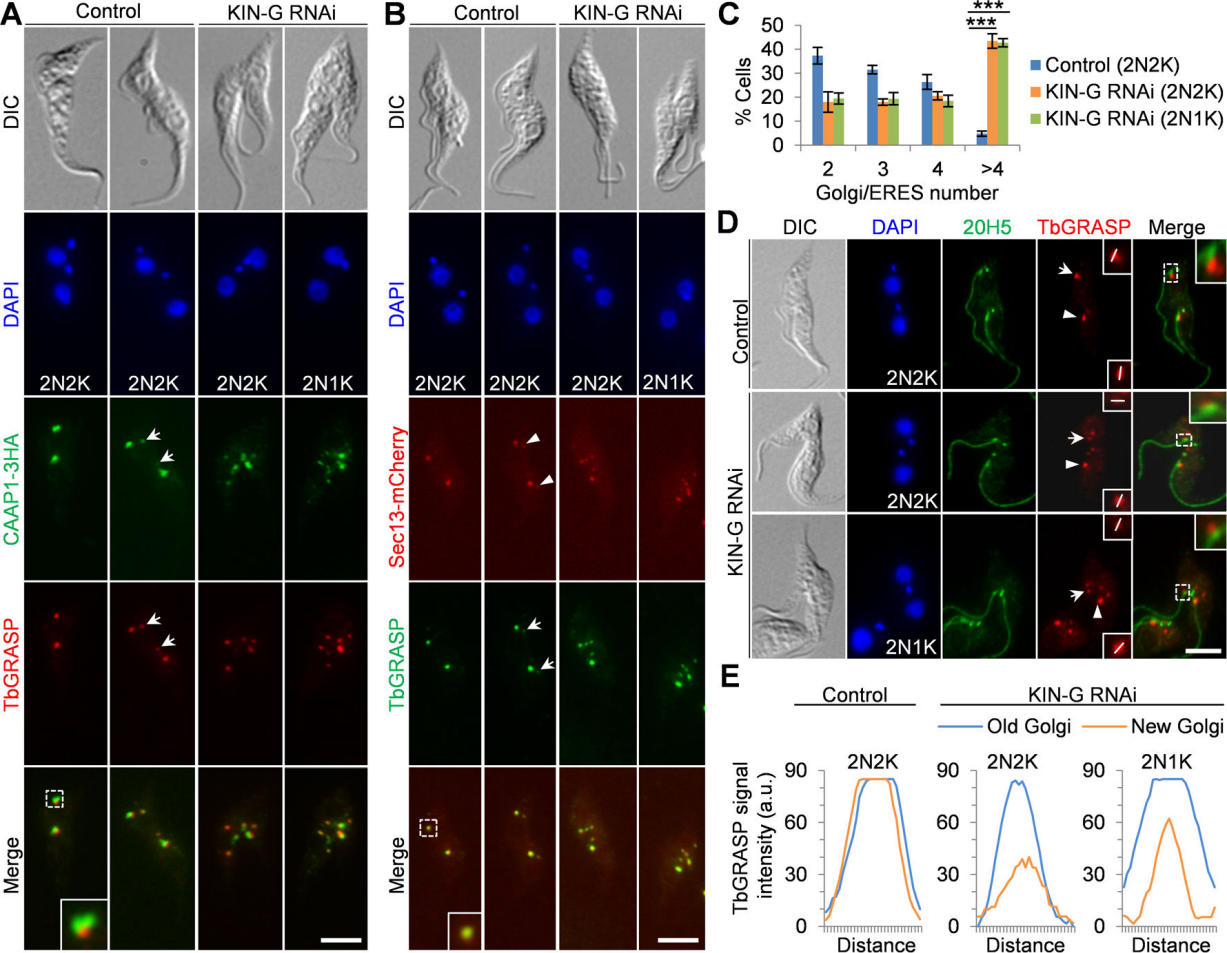
KIN-G is required for Golgi biogenesis. (**A**). Immunostaining of CAAP1 and TbGRASP in control and KIN-G RNAi cells. CAAP1 was endogenously tagged with a triple HA epitope and detected by the FITC-conjugated anti-HA antibody, and TbGRASP was detected by the anti-TbGRASP antibody. The inset shows the 3× zoom-in view of the co-localization of CAAP1 and TbGRASP. Arrows indicate the smaller Golgi in the control cell. Scale bar: 5 µm. (**B**). Immunofluorescence microscopic analysis of Sec13-mCherry and TbGRASP in control and KIN-G RNAi cells. Sec13 was endogenously tagged with mCherry, and TbGRASP was detected by the anti-TbGRASP antibody. The inset shows the 3× zoom-in view of the co-localization of Sec13 and TbGRASP. Arrows indicate the smaller Golgi in the control cell, and arrowheads indicate the smaller ERES in the control cell. Scale bar: 5 µm. (**C**). Quantitation of Golgi and ERES numbers before and after KIN-G RNAi. All of the TbGRASP-labeled, centrin arm-associated and non-associated Golgi were counted. 100 cells each were counted, and error bars indicate SD from three independent experiments. ***P < 0.001. (**D**). Effect of KIN-G RNAi on the association of Golgi with the centrin arm. Centrin arm was labeled by the 20H5 antibody, and Golgi was labeled by the anti-TbGRASP antibody. Arrows indicate the new centrin arm-associated Golgi, and arrowheads indicate the old centrin arm-associated Golgi. The insets in the merged channel show the 3× zoom-in view of the association of the new Golgi with the new centrin arm. The insets in the TbGRASP channel indicate the 3× zoom-in view of the TbGRASP signal at the new and old Golgi, and the white line indicates the transect for quantitative measurement of TbGRASP signal shown in panel E. Scale bar: 5 µm. (**E**). Quantitation of TbGRASP signal intensity in the new and old Golgi that associate with the centrin arm in control and KIN-G RNAi cells presented in panel D.

Knockdown of KIN-G had a surprising effect on CAAP1, by causing the emergence of multiple (>4) CAAP1 fluorescence foci in bi-nucleated cells after RNAi induction for 72 h (Fig. 5A). The recent TrypTag project suggests that CAAP1 localizes to Golgi (30), and our co-immunofluorescence microscopy with the Golgi marker TbGRASP showed that CAAP1 localized in close proximity to TbGRASP (Fig. 5A), indicating that CAAP1 may reside on the periphery of the Golgi apparatus. Strikingly, the knockdown of KIN-G caused similar effects on TbGRASP and the Sec13-marked ERES (ER exit site), a specialized ER zone for cargo transport from the ER to the Golgi (31), producing cells containing multiple (>4) TbGRASP and ERES foci (Fig. 5A and B). In wild-type trypanosome cells, one or two smaller Golgi structures are often detected at the nearby locations of the Golgi (7, 9), and we also detected such smaller Golgi (weaker TbGRASP signal) in control cells (Fig. 5A, arrows), each of which associated with a smaller ERES (Fig. 5B, arrowheads). Knockdown of KIN-G reduced the number of bi-nucleated cells containing two, three, or four Golgi, but resulted in a significant increase in the number of bi-nucleated cells containing more than four Golgi (Fig. 5C). The majority of the Golgi detected in the KIN-G RNAi cells was smaller or had weaker TbGRASP signal (Fig. 5A and B). These results suggest that KIN-G is required for Golgi biogenesis. Similar effects on Golgi biogenesis were also observed in TbPLK RNAi cells (9), suggesting that KIN-G and TbPLK may function in the same pathway to regulate Golgi biogenesis.

In the procyclic form of trypanosomes, the Golgi is located next to the centrin arm, and the duplication of the Golgi depends on the centrin-arm protein TbCentrin2 (8). It was thus proposed that the centrin arm either determines where the new Golgi is assembled or regulates the size of the growing Golgi (8, 9). Since knockdown of KIN-G impaired the integrity of the new centrin arm (Fig. 4), we tested whether the shorter new centrin arm in KIN-G RNAi cells still associates with a Golgi by performing co-immunofluorescence microscopy with the 20H5 antibody to label the centrin arm and the anti-TbGRASP antibody to label the Golgi. In control cells, the two centrin arm-associated Golgi had similar TbGRASP signal intensity (Fig. 5D and E). In KIN-G RNAi cells, however, the new centrin arm-associated Golgi had weaker TbGRASP signal than the old centrin arm-associated Golgi (Fig. 5D and E), and the other Golgi that did not associate with the centrin arms appeared to have weaker TbGRASP signal (Fig. 5D). These results demonstrated that KIN-G knockdown impaired the association of Golgi with the new centrin arm, and suggest that this association may facilitate Golgi biogenesis or maturation.

### CAAP1 is required for Golgi biogenesis and maintains Golgi-centrin arm association

**Fig 6:**
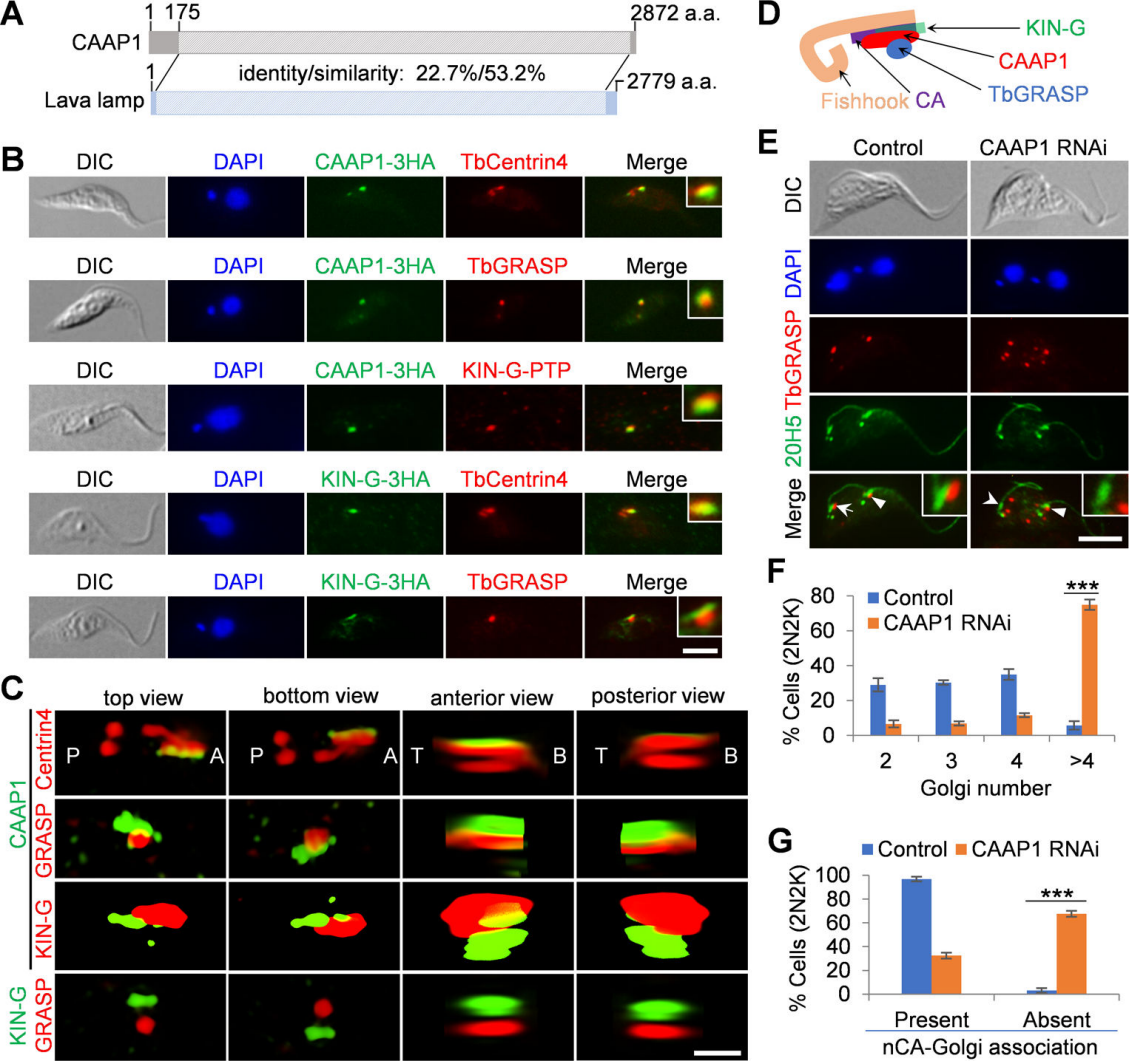
CAAP1 is required for Golgi biogenesis. (**A**) Sequence comparison between CAAP1 and the *Drosophila* Golgi protein Lava Lamp. (**B**) Co-immunofluorescence microscopy to detect the relative localization among CAAP1, TbCentrin4, KIN-G, and TbGRASP. CAAP1 was endogenously tagged with a triple HA epitope and KIN-G was tagged with either a triple HA or a PTP epitope. TbCentrin4 and TbGRASP were detected by their respective antibody. The insets show the 3× zoom-in view of the co-localization between proteins. Scale bar: 5 µm. (**C**) 3D-SIM super-resolution microscopic analysis of the co-localization among 3HA-taagged CAAP1, TbCentrin4, PTP-tagged KIN-G, and TbGRASP. P: posterior; A: anterior; T: top; B: bottom. Scale bar: 0.5 µm. (**D**) Schematic illustration of the relative locations of CAAP1 and KIN-G to the centrin arm and the Golgi. (**E**) Effect of CAAP1 RNAi on Golgi biogenesis. Cells were co-immunostained with the anti-TbGRASP antibody and the 20H5 antibody. The insets show the 3× zoom-in view of the new centrin arm and its associated Golgi. Arrow indicates the new centrin arm-associated Golgi, open arrowhead indicates the new centrin arm without associated Golgi, and solid arrowheads indicate the old centrin arm-associated Golgi. Scale bar: 5 µm. (**F**) Counting of Golgi number in control and CAAP1 RNAi cells. All of the TbGRASP-labeled, centrin arm-associated and non-associated Golgi in the bi-nucleated cells were counted. 100 cells each were counted, and error bars indicate SD from three independent experiments. ***P < 0.001. (**G**). Counting of bi-nucleated cells with (present) or without (absent) new centrin arm-associated Golgi in control and CAAP1 RNAi cells. 100 cells each were counted, and error bars indicate SD from three independent experiments. ***P < 0.001.

The almost identical effects exerted by KIN-G RNAi on CAAP1 and TbGRASP prompted us to analyze the potential structure and function of CAAP1. The CoCOPRED program (32) predicted that CAAP1 contains mostly coiled-coil motifs throughout the entire length of the protein (Fig. S1A), and FASTA protein similarity search (33) identified CAAP1 as a close homolog of a peripheral Golgi protein named Lava Lamp in *Drosophila* (34, 35), with an overall sequence identity/similarity of ~22.7%/53.2% across ~94% of the length of both proteins (Fig. 6A). Lava Lamp is a coiled-coil motif-containing protein and forms a scaffold with the Golgi protein Spectrin to mediate the association of Golgi with microtubules for Golgi movement during *Drosophila* cellularization (34, 35). CAAP1 was localized to the periphery of the Golgi toward the side of the hook complex (Fig. 5A); thus, it may be located between the hook complex and the Golgi. To determine the locations of CAAP1 and KIN-G relative to the centrin arm and the Golgi, we performed co-immunofluorescence microscopy (Fig. 6B). CAAP1 appeared to be located between the centrin arm and the Golgi, overlapping partly with both the centrin arm and the Golgi (Fig. 6B). CAAP1 also co-localized with KIN-G, but it extended beyond KIN-G toward the Golgi (Fig. 6B). KIN-G co-localized mostly with the centrin arm, albeit extending beyond the distal tip of the centrin arm, and localized next to the Golgi (Fig. 6B). The relative localizations of CAAP1, TbCentrin4, TbGRASP, and KIN-G were further confirmed by 3D-SIM super-resolution microscopy, although it was shown that KIN-G and the Golgi are not associated directly (Fig. 6C). Nonetheless, KIN-G appears to reside on the centrin arm, whereas CAAP1 appears to reside in between the centrin arm and the Golgi by bridging the two structures (Fig. 6D). It is possible that CAAP1 may act as a scaffold, as is its *Drosophila* counterpart Lava Lamp, for certain Golgi functions that remain to be explored in trypanosomes, whereas KIN-G either recruits or maintains CAAP1 at the proximity of the centrin arm for the latter to fulfil its function.

We next investigated the potential function of CAAP1 in Golgi biogenesis in trypanosomes. Knockdown of CAAP1 resulted in moderate growth defects (Fig. S1B and C), and immunofluorescence microscopy with the anti-TbGRASP antibody showed that knockdown of CAAP1 caused a significant increase in the number of Golgi, increasing bi-nucleated cells containing more than four Golgi from ~6% to ~75% after CAAP1 RNAi induction for 72 h (Fig. 6E and F), suggesting that CAAP1 is required for Golgi biogenesis. In addition, none of the anti-TbGRASP-labeled Golgi was found to associate with the new centrin arm in ~68% of the CAAP1 RNAi cells induced for 72 h (Fig. 6E and G). This result suggests that CAAP1 is required for maintaining the association of Golgi with the new centrin arm.

### KIN-G localization depends on the centrin arm, and CAAP1 localization requires KIN-G

**Fig 7:**
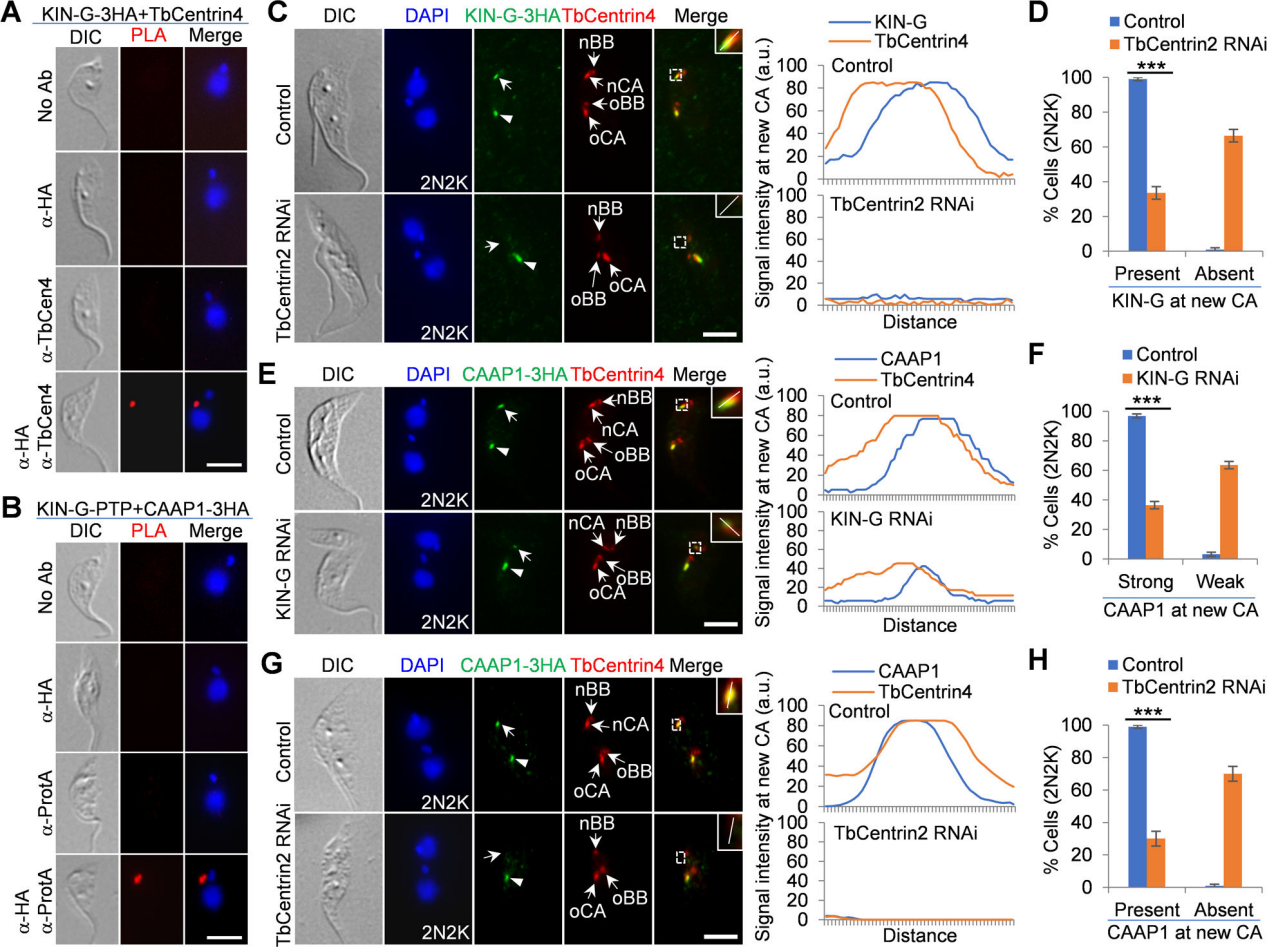
KIN-G localization depends on the centrin arm, and CAAP1 localization requires KIN-G. (**A**) PLA to detect the *in vivo* interaction between KIN-G-3HA and the centrin arm protein TbCentrin4 in trypanosomes. Scale bar: 5 µm. (**B**) PLA to detect the *in vivo* interaction between KIN-G-PTP and CAAP1-3HA in trypanosomes. Scale bar: 5 µm. (**C**) Effect of centrin arm disassembly by TbCentrin2 knockdown on KIN-G localization. Arrows indicate the KIN-G signal at the new centrin arm, and arrowheads indicate the KIN-G signal at the old centrin arm. The insets show the 3× zoom-in view of the co-immunofluorescence signal of KIN-G-3HA and TbCentrin4 at the new centrin arm, and the white line indicates the transect of KIN-G and TbCentrin4 signal for quantitative measurement shown on the right. nBB: new basal body; oBB: old basal body; nCA: new centrin arm; oCA: old centrin arm. Scale bar: 5 µm. (**D**) Quantitation of KIN-G localization at the new centrin arm in bi-nucleated control and TbCentrin2 RNAi cells. 100 cells each were counted, and error bars indicate SD from three independent experiments. ***P < 0.001. (**E**) Effect of KIN-G knockdown on the localization to CAAP1. Arrows indicate the CAAP1 signal at the new centrin arm, and arrowheads indicate the CAAP1 signal at the old centrin arm. The insets show the 3× zoom-in view of the co-immunofluorescence signal of CAAP1-3HA and the new centrin arm, and the white line indicates the transect for quantitative measurement of CAAP1 and TbCentrin4 signal shown on the right. Scale bar: 5 µm. (**F**) Quantitation of CAAP1 localization at the new centrin arm in bi-nucleated control and KIN-G RNAi cells. 100 cells each were counted, and error bars indicate SD from three independent experiments. ***P < 0.001. (**G**) Effect of centrin arm disassembly by TbCentrin2 RNAi on CAAP1 localization at the new centrin arm. Arrows indicate the CAAP1 signal at the new centrin arm, and arrowheads indicate the CAAP1 signal at the old centrin arm. The insets show the 3× zoom-in view of the co-immunofluorescence signal of CAAP1-3HA and TbCentrin4 at the new centrin arm, and the white line indicates the transect of CAAP1 and TbCentrin4 signal for quantitative measurement shown on the right. Scale bar: 5 µm. (**H**) Quantitation of CAAP1 localization at the new centrin arm in bi-nucleated control and TbCentrin2 RNAi cells. 100 cells each were counted, and error bars indicate SD from three independent experiments. ***P < 0.001.

Since KIN-G localizes to the centrin arm, we asked whether KIN-G interacts with the centrin arm. Using TbCentrin4 as a centrin arm marker, we performed the proximity ligation assay (PLA), which showed a positive signal in the centrin arm region (Fig. 7A), suggesting the association of KIN-G with the centrin arm through TbCentrin4. As KIN-G partly co-localizes with CAAP1, we asked whether they form a complex at the centrin arm region. PLA was performed to detect the potential proximal interaction between KIN-G and CAAP1, and the positive signal was detected in the centrin arm region (Fig. 7B), suggesting that KIN-G and CAAP1 are located in such a close proximity that they may form a complex. These results established the close association between the centrin arm and Golgi through KIN-G-mediated association with TbCentrin4 and CAAP1.

As KIN-G associates with the centrin arm, we asked whether KIN-G localization to the centrin arm depends on the assembly/integrity of the centrin arm. We tested this possibility using TbCentrin2 RNAi cells because TbCentrin2 knockdown completely disrupts the formation of the new centrin arm (6). As reported previously (6), the knockdown of TbCentrin2 eliminated the TbCentrin4-labeled new centrin arm (Fig. 7C). This inhibition of new centrin arm formation by TbCentrin2 knockdown caused the complete depletion of KIN-G from the new centrin arm in ~66% of the bi-nucleated cells (Fig. 7C and D). Furthermore, since KIN-G associates with CAAP1, we asked whether CAAP1 localization to the centrin arm region requires KIN-G. Knockdown of KIN-G resulted in the reduction of CAAP1 fluorescence intensity at the new centrin arm in ~64% of the bi-nucleated cells (Fig. 7E and F). Finally, because KIN-G localization depends on the centrin arm and CAAP1 localization depends on KIN-G, we hypothesized that CAAP1 localization also depends on the centrin arm. To test this, we examined CAAP1 localization in TbCentrin2 RNAi cells, and the results showed that inhibition of the assembly of the new centrin arm by TbCentrin2 knockdown eliminated CAAP1 from the new centrin arm in ~70% of the bi-nucleated cells (Fig. 7G and H). Altogether, these results demonstrated that the association of KIN-G with the centrin arm recruits KIN-G and that the association of CAAP1 with KIN-G recruits CAAP1. Thus, KIN-G appears to function to bridge the centrin arm and the Golgi through its association with CAAP1.

### KIN-G function requires motor activity

**Fig 8:**
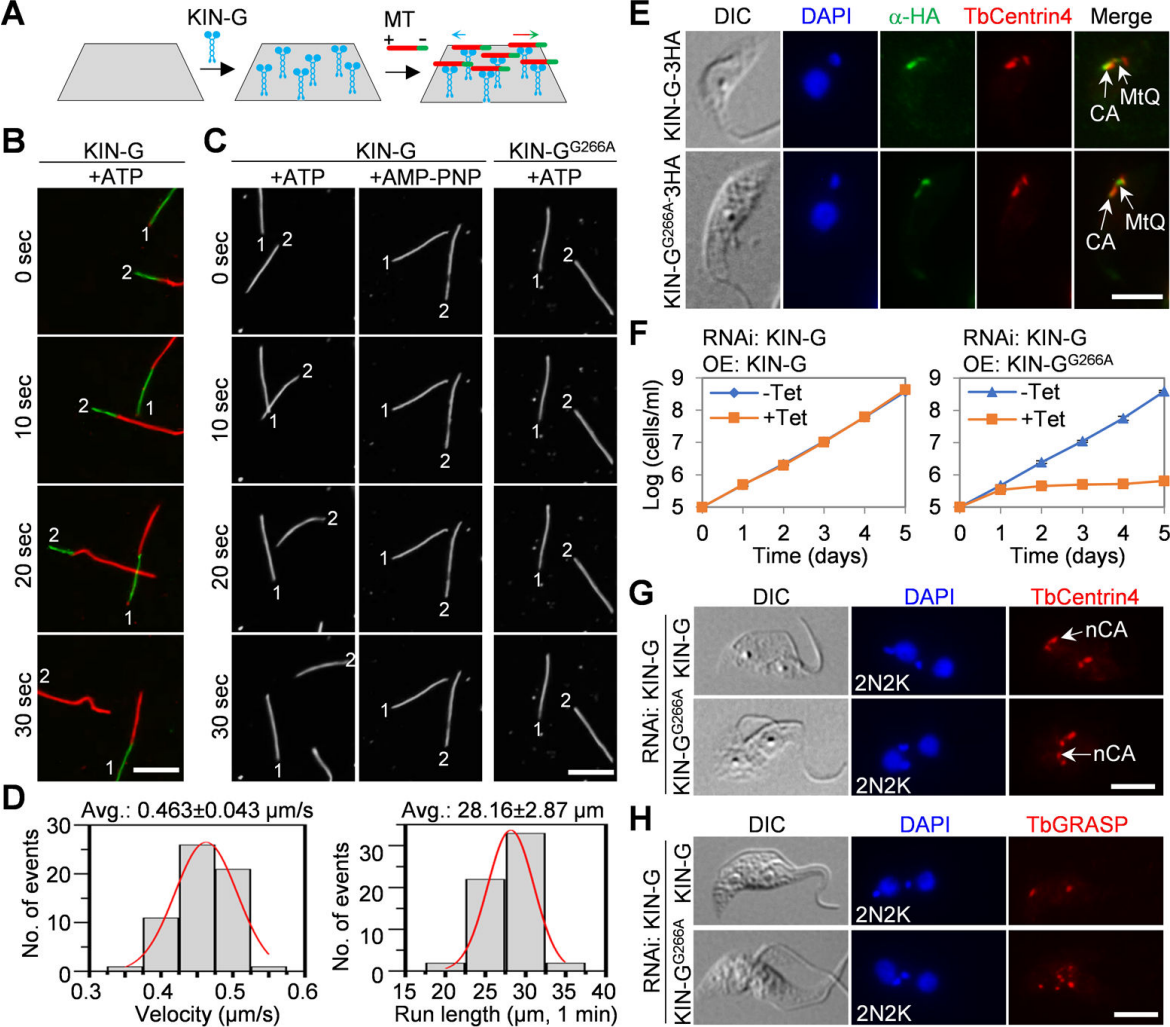
KIN-G is a microtubule plus end-directed motor protein and its motor activity is required for cellular functions. (**A**) Schematic drawing of microtubule gliding assay using purified recombinant KIN-G proteins and *in vitro* assembled microtubules. The green arrow indicates the direction of microtubule movement, whereas the blue arrow indicates the direction of KIN-G movement. (**B**) Microtubule gliding assay for KIN-G using green fluorescence-labeled minus ends of microtubules. (**C**) Microtubule gliding assays for wild-type KIN-G in the presence of ATP or non-hydrolyzable ATP analog AMP-PNP and for the KIN-G^G266A^ mutant in the presence of ATP. (**D**) Velocity and run length (per 1 min) for wild-type KIN-G in the presence of ATP. Avg: average. (**E**) Subcellular localization of ectopic recoded KIN-G and KIN-G^G266A^ tagged with a C-terminal triple HA epitope. CA: centrin arm; MtQ: microtubule quartet. Scale bar: 5 µm. (**F**) Growth curves of KIN-G RNAi cells expressing RNAi-resistant KIN-G or KIN-G^G266A^. OE: ectopic overexpression. (**G**) Effect of expressing RNAi-resistant KIN-G or KIN-G^G266A^ in KIN-G RNAi cells on the integrity of the centrin arm, which was labeled with the anti-TbCentrin4 antibody. nCA: new centrin arm. Scale bar: 5 µm. (**H**) Effect of expressing RNAi-resistant KIN-G or KIN-G^G266A^ in KIN-G RNAi cells on the biogenesis of Golgi, which was labeled with the anti-TbGRASP antibody. Scale bar: 5 µm.

Using recombinant wild-type and mutant KIN-G proteins expressed and purified from bacteria (Fig. S1D), we performed *in vitro* microtubule gliding assay to test the motor activity of KIN-G. To this end, a flow cell was assembled, and purified histidine-tagged KIN-G protein was settled onto the glass coverslip (Fig. 8A). Subsequently, the assembled microtubules were added into the flow cell (Fig. 8A), and the motility of KIN-G along the microtubule was recorded under a fluorescence microscope. Wild-type KIN-G had rigorous motility toward the plus ends of the microtubules in the presence of ATP (Fig. 8B; Movies S1 and S2). However, KIN-G lost motility in the presence of the non-hydrolyzable ATP analog AMP-PNP, and mutation of the conserved glycine residue (Gly-266) in the Switch II domain of the nucleotide-binding motif (Fig. 1A) disrupted the motility (Fig. 8C; Movies S3 and S4). Wild-type KIN-G protein has an average speed of ~0.46 µm/s and travels an average distance of ~28 µm in 1 min (Fig. 8D). These results demonstrated that KIN-G is an ATP-dependent plus end-directed motor protein.

We next tested whether the motor activity of KIN-G is required for KIN-G function in trypanosomes. We ectopically overexpressed a recoded wild-type and G266A mutant of KIN-G in the KIN-G RNAi cell line. We first examined whether motor activity is required for the localization of KIN-G. Immunofluorescence microscopy showed that the ectopic KIN-G was highly enriched at the centrin arm, with a small amount of the protein localized to the proximal end of the MtQ (Fig. 8E). The KIN-G^G266A^ mutant was, however, highly enriched at the proximal region of the MtQ, with a small amount of the mutant protein localized to the centrin arm (Fig. 8E). These results suggest that the immotile KIN-G mutant was somewhat trapped at the proximal region of the MtQ, preventing its movement to the nearby centrin arm structure, where KIN-G promotes hook complex assembly and Golgi biogenesis. Ectopic expression of RNAi-resistant KIN-G in KIN-G RNAi cells restored trypanosome growth (Fig. 8F), but expression of the RNAi-resistant KIN-G^G266A^ mutant in KIN-G RNAi cells failed to rescue the growth defects, demonstrating that the motor activity of KIN-G is required for KIN-G function. It should be noted that KIN-G RNAi cells expressing KIN-G^G266A^ had stronger growth defects than KIN-G RNAi alone (Fig. 2B and 8F), which could be attributed to the additive effects from RNAi and dominant-negative mutation of KIN-G. Finally, to test whether the motor activity of KIN-G is required for KIN-G’s function in promoting hook complex assembly and Golgi biogenesis, we immunostained cells with the 20H5 antibody to label the centrin arm structure and the anti-TbGRASP antibody to label the Golgi apparatus. In the KIN-G RNAi cells expressing the KIN-G^G266A^ mutant, the new centrin arm was shorter than that in the non-induced control cells (Fig. 8G), and multiple (>4) Golgi were detected (Fig. 8H), in striking contrast to that in the KIN-G RNAi cells expressing the wild-type KIN-G protein (Fig. 8G and H). These results demonstrated that the motor activity of KIN-G is required for KIN-G’s function in promoting centrin arm assembly and Golgi biogenesis.

## DISCUSSION

The trypanosome genome lacks some of the evolutionarily conserved kinesins, but it contains many orphan kinesins and kinetoplastid-specific kinesins, some of which might play roles that either compensate for the absence of certain conserved kinesins or are specific to trypanosome development. In this report, we demonstrated that the orphan kinesin KIN-G plays trypanosome-specific functions by promoting the assembly of the hook complex, a trypanosome-specific cytoskeletal structure essential for FAZ elongation and flagellum positioning (5), and regulating Golgi biogenesis. KIN-G localizes to the distal part of the centrin arm (Fig. 1), adding it to the group of orphan and kinetoplastid-specific kinesins possessing distinct subcellular localizations and diverse cellular functions in *T. brucei*. KIN-G is the only kinesin that localizes to the centrin arm and is the first kinesin discovered to promote the biogenesis of the hook complex and the Golgi in *T. brucei*.

The primary function for KIN-G is likely to regulate the integrity of the centrin arm and, hence, the assembly of the hook complex because KIN-G is enriched at the centrin arm (Fig. 1). This notion is supported by the results obtained from the knockdown of KIN-G (Fig. 4). Depletion of KIN-G reduced the length of the new centrin arm (Fig. 4A through C) and caused the loss of the shank part of the fishhook (Fig. 4D and E). Consequently, this impairment of hook complex integrity disrupted the localization of the hook complex-associated TbSmee1 in the new hook complex (Fig. 4F and G). As KIN-G associates with the centrin arm protein TbCentrin4 (Fig. 7), it is possible that KIN-G promotes centrin arm assembly by maintaining TbCentrin4 to form an intact centrin arm. It appears that an intact centrin arm is necessary to maintain the integrity of the fishhook structure of the hook complex and, consequently, a reduction in the length of the centrin arm eliminated the shank part of the fishhook. KIN-G may also transport other hook complex-associated proteins that are involved in hook complex assembly. Finally, because KIN-G is a microtubule plus end-directed motor protein and the immotile KIN-G^G266A^ mutant localizes to the proximal region of the MtQ (Fig. 8), we postulate that KIN-G binds the proximal ends (or the minus ends) of the MtQ, travels toward the plus ends of MtQ, and is detained at the centrin arm by associating with the centrin arm protein TbCentrin4, where KIN-G promotes hook complex assembly by maintaining its integrity.

The inhibitory effect of KIN-G knockdown on the elongation of the new FAZ filament and the positioning of the new flagellum (Fig. 3) is likely attributed to the impairment of hook complex integrity (Fig. 4), as having been demonstrated previously (5, 6, 36). The proximal end of the FAZ filament is embedded within the hook complex (1), and the assembly of the FAZ filament occurs at its proximal end (37, 38). It is possible that the hook complex may act as a hub for the assembly of the FAZ filament. Knockdown of KIN-G produced cells with reduced length of the new FAZ filament (Fig. 3E and F), suggesting that depletion of KIN-G slowed down FAZ assembly. The FAZ in *T. brucei* is required for flagellum attachment to the cell body (27, 39, 40), and defective assembly of the FAZ also causes flagellum mispositioning (27, 37). In this scenario, the mis-positioning of the newly assembled flagellum in KIN-G RNAi cells (Fig. 3A through D) is apparently attributed to the defective FAZ elongation (Fig. 3E and F). Hence, the observed defects in FAZ elongation and flagellum positioning in KIN-G RNAi cells are the secondary effects caused by the impaired assembly of the hook complex.

The requirement of the centrin arm for Golgi biogenesis in *T. brucei* was originally discovered in TbCentrin2 knockdown cells, and it was suggested that the centrin arm determines the location where new the Golgi is assembled (8). KIN-G knockdown also impaired Golgi biogenesis (Fig. 5); however, the effect of KIN-G RNAi on Golgi biogenesis is opposite to that of TbCentrin2 RNAi. The latter inhibited the assembly of new Golgi (8), whereas KIN-G RNAi produced multiple smaller Golgi, ranging from 5 to 8 Golgi in one cell (Fig. 5), similar to TbPLK RNAi (9). The underlying mechanisms for such differences are unclear. Nonetheless, the results obtained from KIN-G RNAi suggest that malformation of the new centrin arm impairs Golgi biogenesis by producing a smaller Golgi that still associates with the mal-formed new centrin arm and many other smaller Golgi that do not associate with the centrin arm (Fig. 5D). Smaller Golgi that do not associate with the centrin arm are also observed in wild-type cells of the procyclic trypanosomes, especially during late mitotic stages, and they disappear during cytokinesis, being either disassembled or integrated into the centrin arm-associated Golgi (7). It is possible that these smaller Golgi may serve as reservoirs of Golgi components for the biogenesis of new Golgi. Hence, when Golgi biogenesis is impaired, these smaller Golgi are massively accumulated.

The mechanistic role of KIN-G in promoting Golgi biogenesis is exerted, at least partly, through the recruitment of CAAP1 to the centrin arm or the maintenance of CAAP1 at the centrin arm (Fig. 7E and F). Previously, CAAP1 was reported as a centrin arm-localized protein (29), but further examination of its localization relative to the centrin arm and Golgi suggests that it localizes to a region between the two structures, likely by serving as a bridge to connect the two structures (Fig. 6B through D). Furthermore, a sequence homology search identified the *Drosophila* Golgi peripheral protein Lava Lamp as a close ortholog of CAAP1 (Fig. 6A), and RNAi-mediated knockdown of CAAP1 in procyclic trypanosomes demonstrated its essential role in promoting the association of Golgi with the centrin arm and, hence, the biogenesis of the new Golgi (Fig. 6E). Thus, like Lava Lamp, CAAP1 also functions as a scaffold for the association of Golgi with certain cytoskeletal structure, with Lava Lamp anchoring Golgi to microtubules through Dynein (35) and CAAP1 maintaining Golgi association with the centrin arm through KIN-G (Fig. 6E and 7B). Therefore, in addition to its essential role in promoting hook complex assembly, KIN-G also plays an essential role in promoting Golgi biogenesis by recruiting CAAP1 to the centrin arm (Fig. 7E and F). By associating with the centrin arm protein TbCentrin4, KIN-G is detained at the centrin arm, where it recruits CAAP1, which maintains the association of Golgi with the centrin arm to facilitate Golgi biogenesis.

Through *in vitro* microtubule gliding assay, we demonstrated that KIN-G is an ATP-dependent plus end-directed motor protein (Fig. 8A through D) and that its motor activity is required for KIN-G localization to the centrin arm (Fig. 8E). Wild-type KIN-G may initially bind the MtQ at the proximal region and then may travel along the MtQ toward the anterior tip, where its microtubule plus-ends are located, but KIN-G may be detained at the centrin arm, likely through association with TbCentrin4 (Fig. 7). The findings that the G266A mutant lost motility (Fig. 8C) and was enriched at the proximal region of the MtQ (Fig. 8E) suggest that this immotile mutant was unable to travel along the MtQ to reach its destination. It should be noted that a small amount of KIN-G^G266A^ was still detected at the centrin arm region, which could be due to the multimerization of the mutant protein with residual amounts of native KIN-G proteins at the centrin arm. Alternatively, it could be due to the binding of the G266A mutant to the MtQ at the overlapping centrin arm region. Nonetheless, the striking contrast of the localization between wild type and the immotile mutant of KIN-G suggests that the motor motility of KIN-G is required for the enrichment of KIN-G at the centrin arm.

Through genetic complementation, we demonstrated that the loss of motor activity impairs the cellular function of KIN-G in trypanosomes (Fig. 8F through H). The consequence of expression of the immotile G266A mutant on hook complex assembly and Golgi biogenesis could be twofold. First, the loss of enrichment of KIN-G at the centrin arm due to the loss of KIN-G motility might impair the association between the centrin arm and the Golgi, thereby disrupting the assembly of the hook complex and the biogenesis of the Golgi apparatus. In this scenario, KIN-G may play a role as a structural bridge between the two structures. Second, the loss of KIN-G motility might disrupt the delivery of certain KIN-G cargos that are necessary for hook complex assembly and Golgi biogenesis, thus preventing the assembly of the hook complex and the biogenesis of Golgi. In this regard, KIN-G, as a plus end-directed motor protein, functions as a cargo transporter to deliver certain construction components for the two structures. The cargos that KIN-G may transport remain to be identified, but the reduction in TbCentrin4 and CAAP1 protein levels at the new centrin arm after depletion of KIN-G (Fig. 7E and F) suggests that these two proteins are potential KIN-G cargos. In addition, KIN-G may also transport certain Golgi components, including but not limited to TbGRASP, whose abundance was significantly reduced at the new centrin arm region when KIN-G was depleted (Fig. 5D and E). Whether KIN-G transports additional cargo remains to be explored.

In summary, we have discovered a novel function for the orphan kinesin KIN-G in regulating hook complex assembly and Golgi biogenesis, and we also identified CAAP1 as a Golgi scaffold protein that promotes the association of Golgi with the centrin arm to facilitate Golgi biogenesis in the procyclic form of *T. brucei*. These findings highlight the unique features in the physical organization of Golgi and the flagellum-associated hook complex and the regulation of the biogenesis process of hook complex and Golgi by a plus end-directed orphan kinesin in procyclic trypanosomes. It should be noted, however, that the function of KIN-G and the regulation of Golgi biogenesis in the bloodstream form of *T. brucei* may be different from that in the procyclic form of *T. brucei* because the interphase cells of the bloodstream form have two Golgi, only one of which associates with the hook complex (41). The exploration of the function of KIN-G in the bloodstream form of *T. brucei* may uncover life cycle-specific features.

## MATERIALS AND METHODS

### Sequence analysis and structural modeling of proteins

Protein homology analysis was performed using the FASTA tool, which provides a heuristic search for homologous proteins against the UniProtKB/Swiss-Protein database (https://www.ebi.ac.uk/Tools/sss/fasta/) (33). Prediction for coiled-coil motifs was performed using the CoCoPRED program (http://www.csbio.sjtu.edu.cn/bioinf/CoCoPRED/index.html) (32). Structural modeling of protein domains was performed using the SWISS-MODEL software (https://swissmodel.expasy.org/) (24, 25). Prediction of protein structure was performed by AlphaFold (22, 23), or the predicted structure was obtained from the AlphaFold protein structure database (https://alphafold.ebi.ac.uk/).

### Trypanosome cell culture and RNAi

The *T. brucei* strain 29–13 (42) was cultured in SDM-79 medium supplemented with 10% heat-inactivated fetal bovine serum (Sigma-Aldrich), 15 µg/mL G418, and 50 µg/mL hygromycin at 27°C, and the *T. brucei* strain Lister427 was grown in SDM-79 medium containing 10% heat-inactivated fetal bovine serum at 27°C. Cells were routinely diluted with fresh medium every 3–4 days whenever the cell density reached 5 × 10^6^ /mL.

To generate the KIN-G RNAi cell line, the CAAP1 RNAi cell line, and the TbCentrin2 RNAi cell line, a 455 bp DNA fragment (nucleotides 341–795) of the KIN-G gene, a 415 bp DNA fragment (nucleotides 4,142–4,556) of the CAAP1 gene, and a 591 bp DNA fragment (nucleotides 1–591) of the TbCentrin2 gene were each PCR amplified from genomic DNA and cloned into the pZJM vector (43), and the resulting plasmids, pZJM-KIN-G, pZJM-CAAP1, and pZJM-TbCentrin2, were used to transfect the *T. brucei* strain 29–13 by electroporation. Transfectants were cultured in SDM-79 medium containing 10% heat-inactivated fetal bovine serum, 2.5 µg/mL phleomycin, 15 µg/mL G418, and 50 µg/mL hygromycin. Successful transfectants were cloned by limiting dilution in a 96-well plate containing the SDM-79 medium with appropriate antibiotics and 20% fetal bovine serum. RNAi was induced with 1.0 µg/mL tetracycline, and cell growth was monitored daily. Three independent clonal RNAi cell lines were chosen for preliminary phenotypic analysis, and they showed almost identical phenotypes. Thus, only one RNAi cell line was used for detailed functional characterization.

### *In situ* epitope tagging of proteins

Epitope tagging of KIN-G, TbSmee1, CAAP1, and Sec13 (Tb927.10.14180) from their respective endogenous locus was carried out using the PCR-based method (44). For KIN-G and CAAP1 co-localization and proximity ligation assay, KIN-G was tagged with a C-terminal triple HA epitope or PTP epitope, and CAAP1 was tagged with a triple HA epitope in the same cell line. Transfectants were selected with appropriate antibiotics, and clonal cell lines were obtained by limiting dilution in a 96-well plate, as described above.

### Immunofluorescence microscopy

To prepare trypanosome cytoskeletons for fluorescence microscopy, cells were washed once with PBS, adhered onto glass coverslips, and treated with 1% Nonidet-P40 in PEME buffer (100 mM PIPES, pH6.9, 2 mM EGTA, 1 mM MgSO4, and 0.1 mM EDTA) for 2 seconds at room temperature, and then fixed with cold methanol at −20°C for 30 min. To prepare intact trypanosome cells for immunofluorescence microscopy, cells were washed once with PBS, settled onto glass coverslips, fixed with methanol at −20°C for 30 min, and rehydrated with PBS at room temperature. Cytoskeletons or intact cells on the glass coverslips were then blocked with 3% BSA in PBS at room temperature for 1 h, and incubated with the primary antibody at room temperature for 1 h. The primary antibodies used are as follows: FITC-conjugated anti-HA monoclonal antibody (1:400 dilution, Sigma-Aldrich), anti-Protein A polyclonal antibody (1:400 dilution, Sigma-Aldrich), anti-CC2D polyclonal antibody (1:1,000 dilution) (27), 20H5 monoclonal antibody (1:400 dilution) (8), L8C4 (anti-PFR2) monoclonal antibody (1:50 dilution) (28), anti-TbCentrin4/LdCentrin1 polyclonal antibody (1:1,000 dilution), anti-TbMORN1 antibody (1:1,000 dilution) (45), anti-TbGRASP polyclonal antibody (1:400 dilution) (7), and anti-TbBILBO1 antibody (1:400 dilution) (46). Cells were washed with PBS and then incubated with secondary antibodies at room temperature for 1 h. The secondary antibodies used are as follows: FITC-conjugated anti-mouse IgG, Cy3-conjugated anti-rabbit IgG, Cy3-conjugated anti-mouse IgG, and FITC-conjugated anti-rabbit IgG (all from Sigma-Aldrich). Cells were washed three times with PBS, mounted with DAPI-containing VectaShield mounting medium (Vector Labs), and then imaged with the Olympus IX71 fluorescence microscope. Images were acquired using the Slidebook 5 software, and they were cropped and merged using the Photoshop software. Images were only adjusted by changing contrast and/or brightness. To measure protein fluorescence intensity, the ImageJ software was used.

### Three-dimensional structured illumination microscopy super-resolution microscopy

Three-dimensional structured illumination microscopy (3D-SIM) super-resolution microscopy was performed according to our published procedures (36). Cells were settled on no. 1.5 glass coverslips (Globe Scientific Inc., USA), fixed in cold methanol (−20°C) for 30 min, re-hydrated in deionized water for 10 min, and incubated in blocking buffer (3% BSA in PBS) for 20 min at room temperature. Cells were co-immunostained with FITC-conjugated anti-HA monoclonal antibody (Sigma-Aldrich), anti-TbCentrin4/LdCen1 polyclonal antibody (47), anti-TbGRASP polyclonal antibody (7), or anti-Protein A polyclonal antibody (Sigma-Aldrich), and then incubated with Cy3-conjugated anti-rabbit IgG (Sigma-Aldrich). Cells on the coverslip were viewed under the Nikon structured illumination superresolution microscope (Nikon Instruments Inc., Americas). The Z-Stack images were taken with Z-steps of 0.1 µm, and 30 images per Z-section were taken. The acquired images were applied to stack reconstruction, and analyzed by the NIS-Elements AR software.

### Scanning electron microscopy

Scanning electron microscopy was performed essentially as described in our previous publication (48). Trypanosome cells were fixed with 2.5% (vol/vol) glutaraldehyde in the cell culture flask for 2 h at room temperature. Fixed cells were harvested by centrifugation at 750 × *g* for 10 min, washed three times with PBS, and settled onto glass coverslips for 30 min. Cells on the coverslip were then dehydrated in a series of alcohol solutions (30%, 50%, 70%, 90%, and 100%) for 5 min each, and then dried by critical point drying. Subsequently, samples were coated with a 5 nm metal film (Pt:Pd 80:20, Ted Pella Inc.) using a sputter-coater (Cressington Sputter Coater 208 HR, Ted Pella Inc.), and imaged using Nova NanoSEM 230 (FEI). The scanning work distance was set at 5 mm, and the accelerating high voltage was set at 8 kV.

### Proximity ligation assay

PLA to detect the *in vivo* interaction between KIN-G and Tbcentrin4 and between KIN-G and CAAP1 was performed using the Duolink *In Situ* PLA Detection kit (Cat#: DUO92008, Sigma Aldrich). *T. brucei* cells co-expressing KIN-G-PTP and CAAP1-3HA from their respective endogenous locus or expressing KIN-G-3HA from its endogenous locus were adhered onto coverslips and fixed with cold methanol (−20°C) for 30 min. Cells were blocked with the Duolink blocking solution and then incubated with anti-Protein A and anti-HA antibodies (for KIN-G-PTP and CAAP1-3HA interaction) or with anti-HA and anti-TbCentrin4 antibodies (for KIN-G-3HA and TbCentrin4 interaction) for 1 h at room temperature. Cells were washed with Buffer A, and probed with the Duolink *In Situ* PLA Probe anti-Mouse MINUS (#DUO92004, Sigma Aldrich) and the Duolink *In Situ* PLA Probe anti-Rabbit PLUS (#DUO92002, Sigma Aldrich) for 1 h at 37°C in a preheated humid chamber. Cells were then washed with Buffer A again, incubated with the ligation solution, and incubated with the amplification solution in a humidity chamber at 37°C for 100 min. Finally, cells were washed with Buffer B, mounted in the Duolink *In Situ* Mounting Medium, and imaged under an inverted fluorescence microscope. For controls, cells were incubated either with no antibodies or with only one of the two antibodies.

### Expression and purification of recombinant proteins

To express and purify C-terminally hexahistidine-tagged KIN-G and its G266A mutant, the full-length coding sequence of KIN-G was cloned into pET26b, and the resulting plasmid was used to generate the G266A mutant by site-directed mutagenesis. Subsequently, the plasmids were each transformed into the *E. coli* BL21 strain. Expression of recombinant proteins was induced with 0.1 mM isopropyl β-d-thio-galactopyranoside for 5 h at room temperature. Bacteria cells expressing hexahistidine-tagged proteins were lysed by sonication in lysis buffer (50 mM NaH_2_PO_4_, 300 mM NaCl, 10 mM imidazole, pH 8.0). The cell lysate was cleared by centrifugation at 20,000 × *g* for 10 min at 4°C, and then was incubated for 30 min at 4°C with the Chelating Sepharose Fast Flow (GE Healthcare) beads charged with nickel ion. Beads were washed five times with 1 mL wash buffer (50 mM NaH_2_PO_4_, 1.0 M NaCl, 120 mM imidazole, pH 8.0). Recombinant proteins were eluted with elution buffer (50 mM NaH_2_PO_4_, 300 mM NaCl, 250 mM imidazole, pH 8.0) and were concentrated and buffer-exchanged with Amicon Ultra Centrifugal Filters 10K (Millipore).

### Tubulin polymerization and microtubule gliding assay

*In vitro* tubulin polymerization was performed according to our published procedure (49). Briefly, non-labeled porcine brain tubulin (Cytoskeleton, Inc., Cat#: T240-A80, 80 µg) was mixed with rhodamine-labeled porcine brain tubulin (Cytoskeleton, Inc., Cat#: TL590M, 20 µg) or Fluorescent HiLyte 488-labeled porcine brain tubulin (Cytoskeleton, Inc., Cat#: TL488MA, 20 µg) in BRB80-DTT buffer (80 mM Potassium-PIPES, pH 6.8, 1.0 mM MgCl_2_, 1.0 mM EGTA, 1.0 mM DTT) supplemented with 1.0 mM Guanylyl-(α,β)-methylene-diphosphonate (GMP-CPP), and the mixture was incubated at 4°C for 5 min before it was further clarified by centrifugation at 353,000 × *g* at 4°C for 5 min in a TLA120.1 rotor in an ultracentrifuge (Beckman Coulter TL-100). The supernatant was aliquoted, snap-frozen in liquid nitrogen, and stored at −80°C. To generate microtubule seeds, an aliquot of the supernatant was 1:4 diluted with BRB80-DTT buffer to a final concentration of 0.5 mg/mL and was incubated at 37°C for 30 min. Microtubule seeds were pelleted at 353,000 × *g* at 27°C for 5 min, and the pellet was resuspended with BRB80-DTT buffer.

To polymerize microtubules from the microtubule seeds, 20 µg of rhodamine-labeled tubulin was mixed with the microtubule seeds in the presence of 2.0 mM GTP. Microtubules were then successively assembled by incubating at 37°C with 0.1 µM Taxol in BRB80-DTT-GTP buffer (BRB80-DTT buffer plus 1.0 mM GTP) for 20 min, 1 µM Taxol for 10 min, and 10 µM Taxol for 10 min. Assembled microtubules were diluted with 10 µM Taxol in BRB80-DTT-GTP buffer.

To generate polarity-marked microtubules, 80 µg of non-labeled tubulin in 8 µL BRB80 was mixed with 0.9 µL of 10 mM N-ethylmaleimide (NEM, Acros Organics), which causes near-to-complete arrest of microtubule elongation from the minus ends (50), at room temperature for 5 min. The reaction was stopped by adding 1 µL of 200 mM DTT and incubating at 4°C for 15 min. Insoluble aggregates were removed by centrifugation at 353,000 × *g* for 15 min at 4°C. A microtubule elongation mix containing 40 µg non-labeled tubulin, 5 µg rhodamine-labeled tubulin, and 9 µL of NEM-treated tubulin in BRB80-DTT-GTP buffer (total volume 30 µL) was incubated at 37°C for 1 min, followed by the addition of 5 µL of the microtubule seeds made with HiLyte 488-labeled tubulin and incubation at 37°C for 30 min. Microtubules were further polymerized by adding Taxol stepwise, as mentioned above.

Microtubule gliding assay was conducted in a chamber assembled on a glass slide with two double-sided tapes, which were spaced 8 mm apart, and covered with a 22 mm × 30 mm cover glass. Twenty microliters of 1.2 µM purified His-tagged KIN-G in BRB80-DTT-ATP buffer (BRB80-DTT buffer plus 1.0 mM ATP) supplemented with 800 ng/mL BSA was loaded into the chamber and incubated for 3 min to attach His-tagged KIN-G to the cover glass. The chamber was washed twice with 20 µL of BRB80-DTT-ATP buffer and then loaded with 20 µL of 22.5 nM rhodamine-labeled microtubules (or polarity-marked microtubules) in BRB80-DTT-ATP buffer supplemented with 2.25 mg/mL BSA. After a 3-min incubation, the chamber was washed twice with BRB80-DTT-ATP buffer. The slide was immediately observed for microtubule gliding under the Nikon A1 confocal microscope, and images were captured every 5 sec. Microtubule movement in AVI format movie files was generated by ImageJ.

### Ectopic expression of KIN-G and its G266A mutant and genetic complementation

The full-length coding sequence of KIN-G was recoded by replacing the third codon of its amino acids and the resulting recoded KIN-G gene was cloned into pLew100-3HA-PAC vector. The resulting plasmid was then used for mutagenesis to mutate the conserved glycine residue in the switch II motif of the nucleotide-binding motif of the kinesin motor domain. The two plasmids were used to transfect the KIN-G RNAi cell line, and transfectants were selected with 1 µg/mL puromycin in addition to 2.5 µg/mL phleomycin, 15 µg/mL G418, and 50 µg/mL hygromycin. Successful transfectants were further cloned by limiting dilution, as described above. Expression of recoded KIN-G and KIN-G^G266A^ and knockdown of endogenous KIN-G were induced with 1 µg/mL tetracycline. Cell growth was monitored by daily counting of the cells with a hemacytometer.

### Cell counting and statistical analysis

For cell counting, fluorescence microscopic images were taken randomly, and all the cells in the image were counted. Data were obtained from three independent experiments. or statistical analysis, one-way analysis of variance (ANOVA) and two-tailed Student’s *t* test were used.

## SUPPLEMENTAL MATERIAL

Supplemental data mbio.02634-24-s0001.pdf Figure S1 and legends for Movies S1-S4.

Movie S1 mbio.02634-24-s0002.avi Microtubule gliding assay for wild-type KIN-G in the presence of ATP using microtubules assembled with Fluorescence HiLyte 488-labeled microtubule seeds (minus ends of the assembled microtubules) and rhodamine-labeled tubulins.

Movie S2 mbio.02634-24-s0003.avi Microtubule gliding assay for wild-type KIN-G in the presence of ATP using microtubules assembled with rhodamine-labeled tubulins.

Movie S3 mbio.02634-24-s0004.avi Microtubule gliding assay for wild-type KIN-G in the presence of non-hydrolysable ATP analog AMP-PNP using microtubules assembled with rhodamine-labeled tubulins.

Movie S4 mbio.02634-24-s0005.avi Microtubule gliding assay for the KIN-G-G266A mutant in the presence of ATP using microtubules assembled with rhodamine-labeled tubulins.

ASM does not own the copyrights to Supplemental Material that may be linked to, or accessed through, an article. The authors have granted ASM a non-exclusive, world-wide license to publish the Supplemental Material files. Please contact the corresponding author directly for reuse.
