# Supplementary material for: An orphan kinesin in Trypanosoma brucei regulates hook complex assembly and Golgi biogenesis
Source: mBio. 2024 Oct 30;15(12):e02634-24. doi: 10.1128/mbio.02634-24 (PMC11633155; doi:10.1128/mbio.02634-24)
Supplement: Supplemental data — Figure S1 and legends for Movies S1-S4. [file mbio.02634-24-s0001.pdf]

# Supplemental files

## Figure S1

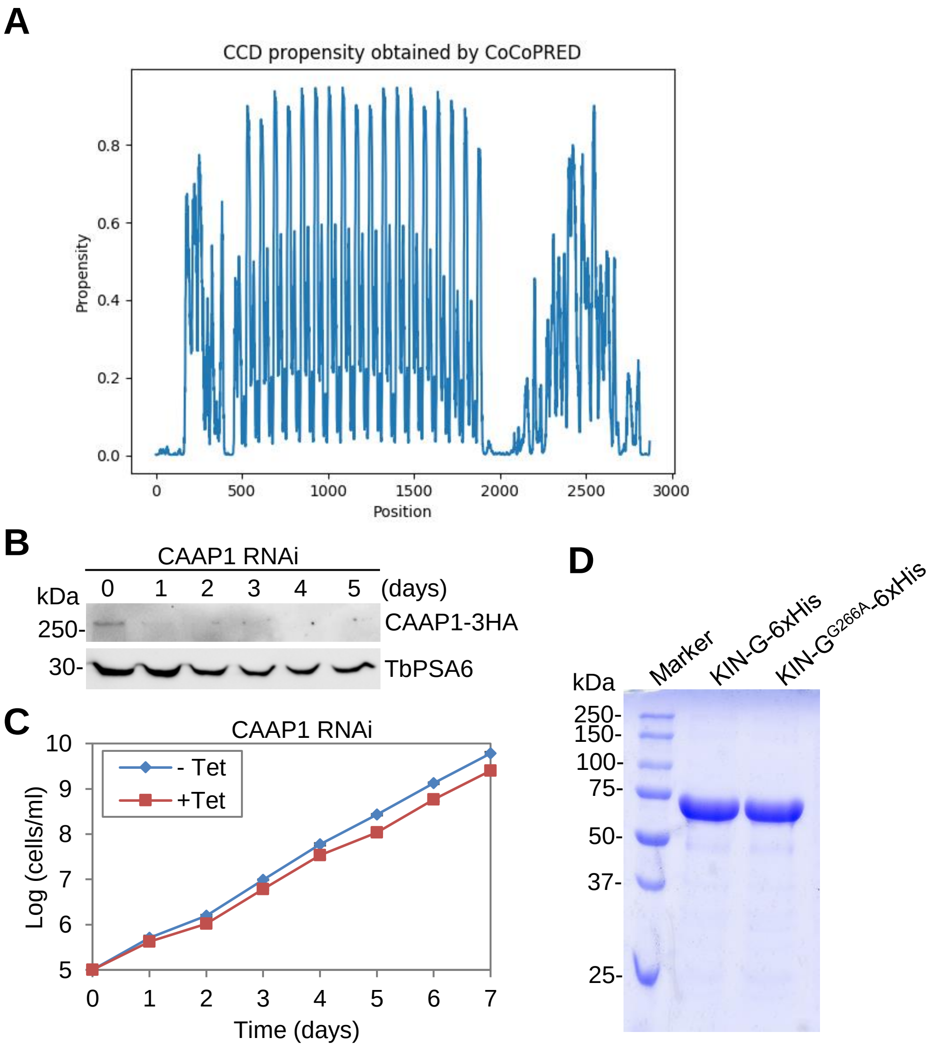


**Figure S1. RNAi-mediated knockdown of CAAP1 in procyclic trypanosomes and purification of wild-type and mutant KIN-G for *in vitro* microtubule gliding assay.** (**A**). Distribution of coiled-coil motifs in CAAP1 predicted by the MultiCoil program. (**B**). Western blotting to monitor the CAAP1 protein level before and after RNAi induction. CAAP1 was endogenously tagged with a triple HA epitope. TbPSA6 serves as a loading control. (**C**). The growth curve of non-induced and RNAi-induced CAAP1 RNAi cell line. (**D**). Expression and purification of KIN-G and KIN-G$^{G266A}$ from bacteria.

**Movie S1**. Microtubule gliding assay for wild-type KIN-G in the presence of ATP using microtubules assembled with Fluorescence HiLyte 488-labeled microtubule seeds (minus ends of the assembled microtubules) and rhodamine-labeled tubulins. The video plays at 5 fps, and images (frames) were taken with a 5 second interval.

**Movie S2**. Microtubule gliding assay for wild-type KIN-G in the presence of ATP using microtubules assembled with rhodamine-labeled tubulins. The video plays at 5 fps, and images (frames) were taken with a 5 second interval.

**Movie S3**. Microtubule gliding assay for wild-type KIN-G in the presence of non-hydrolysable ATP analog AMP-PNP using microtubules assembled with rhodamine-labeled tubulins. The video plays at 5 fps, and images (frames) were taken with a 5 second interval.

**Movie S4**. Microtubule gliding assay for the KIN-G$^{G266A}$ mutant in the presence of ATP using microtubules assembled with rhodamine-labeled tubulins. The video plays at 5 fps, and images (frames) were taken with a 5 second interval.
